# Association Between the *COL5A1* rs12722 Genotype and the Prevalence of Anterior Cruciate Ligament Rupture in Professional Football Players

**DOI:** 10.3390/genes16060649

**Published:** 2025-05-28

**Authors:** Manuel Manchón-Davó, Juan Del Coso, Francisco J. Vera-Garcia, Joaquín González-Rodenas, Aarón Miralles-Iborra, Gil Rodas, Roberto López-Del Campo, Víctor Moreno-Pérez

**Affiliations:** 1 Sports Research Centre, Department of Sport Sciences, Miguel Hernandez University of Elche, 03202 Alicante, Spain; manuel.manchon01@alu.umh.es (M.M.-D.); fvera@umh.es (F.J.V.-G.); aaron.mirallesi@umh.es (A.M.-I.); 2 Sport Sciences Research Centre, Faculty of Education and Sport Sciences and Interdisciplinary Studies, Rey Juan Carlos University, 28942 Fuenlabrada, Spain; joaquin.gonzalez@urjc.es; 3 Institute for Health and Biomedical Research, ISABIAL Foundation, Neurosciences, Miguel Hernández University of Elche, 03010 Alicante, Spain; 4 Medical Department & Barça Innovation Hub, Fútbol Club Barcelona, 08970 Barcelona, Spain; gil.rodas@fcbarcelona.cat; 5 Department of Competitions and Mediacoach, LaLiga, 28043 Madrid, Spain; rlopez@laliga.es; 6 Translational Research Centre of Physiotherapy, Department of Pathology and Surgery, Faculty of Medicine, Miguel Hernandez University, 03550 Alicante, Spain

**Keywords:** knee, knee ligament, genetics, soft-tissue injury, soccer, team sport

## Abstract

Background: Previous studies have tested the association between the *COL5A1* rs12722 polymorphism and the risk of anterior cruciate ligament (ACL) injury. Overall, their results are contradictory because most studies used relatively small samples and data from ACL ruptures during sport activities have been mixed with ruptures suffered in non-sporting contexts. Objective: To examine the association between the *COL5A1* rs12722 polymorphism and the prevalence of ACL rupture in a homogeneous sample of professional male football players. Methods: A total of 268 professional male football players participated in this study. The *COL5A1* rs12722 genotype (CC, CT and TT) was obtained from each player using genomic DNA samples obtained from a buccal swab and measured with PCR RFLP. Players with history of ACL rupture during their professional career were identified by the medical staff of each team. Only ACL injuries obtained during football exposure were considered for this investigation. In this process, we identified 49 ACL ruptures pertaining to 43 players suffered between 2013 and 2024. The situational pattern (i.e., attacking or defending, type of football action, moment of the season, match/training exposure, etc.) was also obtained for each injury. A sub-analysis of non-contact ACL ruptures was conducted, as these injuries are more likely to be influenced by genetic factors. Results: The distribution of genotypes was similar in players with history of ACL rupture (n = 43; CC/CT/TT, 24.4/48.9/26.7%) and with no history of ACL rupture (n = 225; 25.3/49.8/24.9%; *p* = 0.973). Overall, the prevalence of players with history of ACL injury was 16.2% for the whole group of CC players (11 out of 68 players), 16.4% for whole group of CT (22 out of 135 players) and 15.2% for the group of TT players (10 out of 66 players; *p* = 0.973). However, the *COL5A1* rs12722 genotype affected the dominance of the injured leg (*p* = 0.012), the type of action that originated the injury (*p* = 0.047), and the distribution of non-contact ACL injuries depending on the time of the match (*p* = 0.020). Specifically, CC players suffered ruptures predominantly in the dominant leg, when landing or reaching (offensive actions) and in the last 15 min of the match (all *p* < 0.050). On the contrary, TT players had ACL ruptures predominantly in their non-dominant leg, when pressing the opponent (defensive actions) and in the first 15 min of the match (all *p* < 0.050). Conclusion: There was no association between any of the *COL5A1* rs12722 genotypes and the overall prevalence of ACL rupture in professional football players. However, the *COL5A1* rs12722 polymorphism appeared to influence specific characteristics of the injury, such as the type of action leading to the rupture and the timing within the match, suggesting a potential genetic contribution to injury susceptibility.

## 1. Introduction

Anterior cruciate ligament (ACL) rupture represents one of the most disabling injuries in football (soccer). Although it represents only 1.42% of all reported injuries in elite football teams [1], the average recovery time for return-to-play is 222 days [2]. Each season, approximately 11 football players in a major league suffer from ACL ruptures, which averages to 0.55 injuries per team [3]. Unfortunately, the rate of ACL rupture among professional football players increased by 18% from the 1990s to the 2010s [4], likely due to the increasing physical demands of modern football. The negative impact of ACL injuries extends beyond their incidence and the prolonged rehabilitation process as these injuries are associated with an increased risk of reinjury within two years [5,6] and a higher likelihood of developing chronic knee instability and/or articular cartilage damage [7,8]. The frustration players experience from an ACL injury is further intensified by the decline in performance often seen in professional footballers who have suffered this injury [1]. Two years after ACL reconstruction, 90% of football players are still competing at the top level; however, this percentage drops to 60% after five years [9]. his year-by-year decline may be due to the combination of reduced performance following return to play and the natural progression of age. Interestingly, players who remained at the top level five years post-reconstruction were younger at the time of injury [9] suggesting that older players are less likely to regain their pre-injury performance or secure contracts in top-tier teams after a prolonged recovery period. The overall decline in performance likely affects player’s ability to return to peak form, compounding the emotional and physical challenges of recovery from this injury. Professional clubs now recognize the importance of implementing preventive measures to reduce the impact of ACL injuries and protect players’ health. However, the understanding of ACL injury risk factors in football remains incomplete, suggesting that current prevention programs may be suboptimal and need further refinement to be more effective.

Numerous intrinsic and extrinsic risk factors have been identified as contributing to the likelihood of ACL injury [10,11]. However, despite this evidence, the exact etiopathogenesis of ACL rupture has not yet been completely deciphered [11]. Over the last decade, several genes have been analysed for association with ACL rupture [12,13,14], mainly genetic variations in collagen-related genes. Among the different genes codifying subtypes of collagen, the *COL5A1* gene is one of the most investigated with the association of the ACL rupture in athletes [12,14,15,16,17,18,19]. The *COL5A1* gene is located on chromosome 9q34.3, codes for the α1 chain of type V collagen, which is a fibrillar collagen found in ligaments and other soft tissues [15]. The α1 chain of type V supports many tissues in the body, such as tendons, ligaments, and muscles. Within the *COL5A1* gene exists a single nucleotide polymorphism (rs12722 C/T, also known as BstUI) that implies a cytosine-to-thymine transitions. Individuals with the T allele of rs12722 possess higher messenger RNA stability than the C allele [20] and which presumably increases the amount of type V collagen produced in the cell. However, an excessive amount of type V collagen may alter fibrillogenesis processes and overall collagen structure within the tissue [21]. Type V collagen is known to interact with type I collagen to form a basic subunit upon which further collagen molecules build upon. Therefore, altered abundance of type V protein may change the biochemical and thus biomechanical properties of ligaments [22].

Following this hypothesis, previous reports found that athletes with the rs12722 TT genotype of the *COL5A1* showed a trend toward a higher risk of ACL rupture than athletes with the CC genotype [15,17,23,24,25]. However, a lack of influence on the *COL5A1* rs12722 polymorphism on the risk of ACL rupture has also been found in other studies performed with elite athletes [12,16,19], including a recent meta-analysis of studies on the topic [18]. The differences in the outcomes of these investigations might be attributable to the relatively small samples used in most studies, particularly within the group of athletes with ACL ruptures and the variability in the samples catalogued as athletes. Most studies linking the *COL5A1* rs12722 polymorphism to ACL rupture risk in athletes have used mixed samples that include participants from various sports, such as volleyball, basketball, hockey, or samples with active populations [17,23,24,26]. This is a limitation, as the epidemiological characteristics of ACL ruptures can vary significantly across different sports and may also differ from the types of injuries experienced by active individuals who are not engaged in organized sports. Another limitation of current literature is the use of mixed samples of athletes across different performance levels, as well as the lack of detailed information on the situational patterns that lead to ACL ruptures.

To bridge the gap with current literature, the aim of this study was to examine the association between the *COL5A1* rs12722 polymorphism and the prevalence of ACL rupture in a wide and homogeneous sample of professional male football players. We hypothesized that the rs12722 TT genotype would be overrepresented in the sample of players with history of ACL rupture, especially among those with a non-contact ACL injury.

## 2. Materials and Methods

### 2.1. Participants

A total of 268 professional football players from 10 teams competing in the first division of football in Spain (*LaLiga*) volunteered to participate in this investigation. From the total, 227 players were of Caucasian descent, 20 were of African American descent, and 1 was of Asian descent. In the sample, there were 28 goalkeepers, 44 center defenders, 45 full backs, 76 center midfielders, 32 wingers and 43 forwards. Among these players, 43 had a history of ACL rupture as they had been diagnosed at least once with a total ACL tear occurred during football exposure and confirmed by MRI at any point of their professional careers (cases) and 225 players had no history of ACL rupture during their professional careers (controls). An *a priori* sample size calculation estimated a minimum of 46 participants, based on an effect size of w = 0.462. This estimate was derived from a previous study reporting that the CC genotype was significantly underrepresented in the anterior cruciate ligament (ACL) rupture group compared to healthy controls (27.4% vs. 5.6%; odds ratio = 6.6) [23]. T The G*Power software (version 3.1.9, Germany) was used to perform the sample size calculation, assuming that Chi-square (χ^2^) tests and contingency tables would be applied to compare *COL5A1* rs12722 genotype distributions between individuals with and without ACL injury. The analysis was designed to achieve a statistical power of 80% and a significance level (α) of 0.05. Ultimately, we were able to recruit a larger sample than initially estimated, as 10 out of the 20 teams competing in LaLiga agreed to participate in the study. Before the start of this investigation, written informed consent was obtained from all players. The procedures were approved by the institutional Ethics Review Committee (DPC.VMP.01.20) of the Miguel Hernandez University and were conducted in accordance with the consensus statement of the International Federation of Sports Medicine regarding the use of genetic information [27].

### 2.2. Genetic Testing

Genetic data were obtained during the pre-season period of the 2021–2022 season (July–August 2021). A member of the research team visited the teams’ training facilities, explained the study’s objectives, and answered any questions the players had regarding the use of their samples. Following this, all players provided written informed consent. Then, participants completed an ad hoc questionnaire to obtain socio-demographic information and body mass, and height were measured (Radwag, Poland). Then, genomic DNA samples were collected via buccal swabs using a cotton swab. The DNA samples were identified with an alphanumeric code and did not contain any personal information that allowed the identification of the player. At a later stage, genomic DNA was isolated from each sample using standardized protocols [28] and genotyping was conducted in a certified genetics laboratory. Internal controls, including reference samples (blank and negative controls), were used throughout, with contamination monitoring implemented at each step to ensure sample integrity. Specifically, genomic DNA was isolated using an organic-based DNA extraction method adapted to Amicon^®^ Ultra-0.5 mL columns (Sigma-Aldrich, Madrid, Spain), including a final concentration step to 50 µL. The extracted DNA was subsequently subjected to PCR. Positive controls for all genotypes were used from the Mexican branch of CANDELA Consortium. Genotyping of the *COL5A1* polymorphism (c.*267C>T; 3 prime UTR variant-[Sequence Ontology: SO:0001624]) was conducted using a TaqMan single nucleotide polymorphism (SNP) Genotyping Assay (Assay ID: C____370252_20); rs12722 Applied Biosystems) and the reaction was performed in an Applied Biosystems 7500 Fast Real-Time PCR System (Applied Biosystems, Foster City, CA, USA). Genotyping analysis was performed using 7500 Software v2.0.5 (Applied Biosystems). Any samples with ambiguous rs12722 genotype results were reanalyzed to ensure accuracy. To assess reproducibility, 69 samples were randomly selected and genotyped in duplicate, confirming complete concordance between both runs. The laboratory assigned genotype data to anonymized alphanumeric codes, and only the research team held the key linking these codes to individual player data. This process ensured confidentiality and prevented the identification of participants during the genotyping procedures.

### 2.3. History of ACL Rupture During Football Exposure and Mechanism of Injury

**Figure 1:**
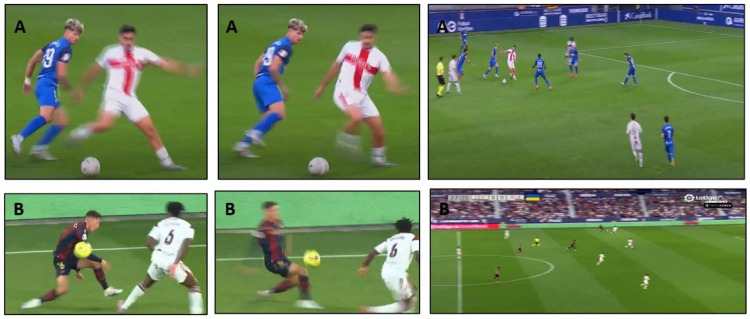
Example of video analysis of two ACL ruptures during match play to categorize situational patterns: (**A**) non-contact ACL rupture of the left leg during a defensive pressing action (player in white T-shirt); (**B**) non-contact ACL rupture of the right leg during an attacking dribbling action (player in black T-shirt).

Players with history of ACL rupture during their professional career were identified by the medical staff of each team. Only injuries obtained during football exposure were considered for this investigation. Then, we compared the data provided by the medical staff with the data provided by the players in the information questionnaire to certify that we had correctly identified all players with a history of ACL rupture (there were no discrepancies). In this process, we identified 49 ACL ruptures that occurred between 2013 and 2021. For the classification of ACL ruptures, the medical staff used an ad hoc questionnaire created by the research group of this study following the classification system developed by the UEFA [29]. The questionnaire contained a clear definition of ACL rupture as any complete tear of the ACL of the knee joint, occurring either isolated or associated with other concomitant injury. All reported injuries had to be diagnosed through physical exams and MRI. All identified ACL ruptures required surgical reconstruction, and they were also confirmed surgically. The classification also included information to determine the limb injured and if it was a first-time injury (first occurrence of ACL rupture, with no prior history of ACL injury in either leg), a second injury in the same leg (ipsilateral re-injury as a subsequent ACL rupture occurring in the same knee that sustained the initial injury, indicating a re-injury of the previously affected ligament) or a second injury in the contralateral leg (an ACL rupture occurring in the opposite knee to the one previously injured, representing a new injury event in the contralateral limb). In addition, the medical staff recorded three categories of ACL rupture depending on the mechanism that led to the injury: (a) non-contact (i.e., injury that did not involve contact with another player or object immediately before or at the same time of the injury), (b) indirect contact (i.e., injury due to external forces that did not directly cause the injury but affected the natural movement process and indirectly led to the injury, such as an upper body contact) and (c) direct contact (i.e., injury due to external forces directly at or adjacent to the injured body site, either by contact with another player or with an object such as the goal or the ball, such tacking or being tackled), following previous recommendations [30]. A sub-analysis of non-contact ACL ruptures was conducted, as these injuries are more likely to be influenced by genetic factors. The dominant leg was defined as the preferred leg used by the player to kick the ball, according to technical and performance data provided by *LaLiga.* The situational pattern, which describes the interaction between the ACL injury action and the football context (i.e., attacking or defending, type of football action, moment of the season, match/training exposure, minutes played up to the moment of the injury, etc.) was also obtained for each injury. When feasible, the medical staff accessed video footage of the injury, as clubs typically maintained video records or obtained them from television broadcasts. The return to play was defined as the time between the day of the injury and the point at which a player was medically cleared and deemed physically and psychologically ready to resume full participation in matches and training without restrictions. A checklist and definition terms used to categorize each ACL rupture can be seen in Table 1. An example of the video-analysis to each rupture to determine the situational patterns can be seen in Figure 1.

### 2.4. Statistical Analysis

To begin the analysis, we assessed whether the distribution of *COL5A1* rs12722 genotypes in our sample conformed to Hardy-Weinberg equilibrium using a chi-square (χ^2^) test. Additionally, a separate χ^2^ test was conducted to compare the genotype frequencies observed in our cohort of football players with those reported for ethnically matched controls from the 1000 Genomes database [31]. Specifically, we compared football players with a Caucasian origin with the rs12722 genotype distribution of the Caucasian-European population (CC, 18.5%; CT, 45.9%; and TT, 35.6%). Likewise, we compared players with an Afro-American origin with the rs12722 genotype distribution of the African population from the 1000 Genomes database (CC, 72.3%; CT, 26.3%; and TT, 1.4%). Descriptive statistics were calculated for players within each genotype as frequencies for categorical variables and as mean ± standard deviation for continuous variables. Differences among genotypes for categorical variables (e.g., ethnicity or field position) were determined with χ^2^ tests. In the case of a significant χ^2^ test, standardized residuals were calculated to identify which genotype had an abnormal distribution. For continuous variables such as age and anthropometric measures, the assumption of normality was assessed using the Kolmogorov–Smirnov test, which confirmed a normal distribution for all variables. Differences across genotype groups were evaluated using one-way analysis of variance (ANOVA). When a significant main effect of genotype was detected (based on the F statistic), Bonferroni-adjusted post hoc comparisons were conducted to determine specific group differences. The threshold for statistical significance was set at *p* < 0.050. All analyses were carried out using SPSS Statistics software (version 28.1; IBM Corp., Armonk, NY, USA).

## 3. Results

### 3.1. Sociodemographic Variables and Field Position

**Figure 2:**
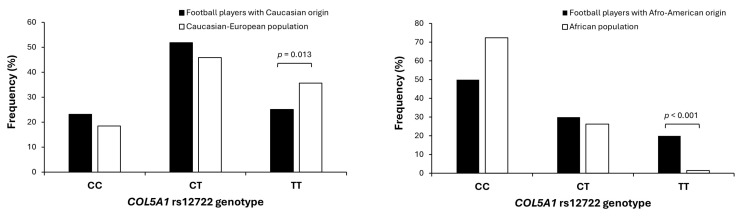
Distribution of *COL5A1* rs12722 genotypes in a sample of 247 football players of Caucasian origin and 20 football players of African-American origin, compared with the genotype distribution of ethnically matched controls from the 1000 Genomes database [31].

Genotyping success was 100%, with the following genotype distribution for the whole sample of professional football players: CC, 25.4%; CT, 50.0%; and TT, 24.6% (Table 2). The genotype distribution followed HWE at *p* = 0.999. Age, body mass and body mass index were comparable in all genotypes although CC players were taller than CT players (*p* = 0.013). The distribution of players according to their ethnicity was affected by the *COL5A1* rs12722 genotype (*p* = 0.037). In the group of players with a Caucasian origin, there was a lower frequency of CC players with respect to the group of players with an Afro-American origin (*p* < 0.050). Additionally, the sample of professional football players with a Caucasian origin presented a lower frequency of TT than in the Caucasian-European population described in the 1000 Genomes database (*p* = 0.013, Figure 2). On the contrary, the sample of professional football players with Afro-American origin presented a higher frequency of TT with respect to the African population described in the 1000 Genomes database (*p* < 0.001, Figure 2). Last, the distribution of players according to their position in the field was not affected by the *COL5A1* rs12722 genotype.

### 3.2. History of ACL Rupture and Its Mechanism of Injury

**Figure 3:**
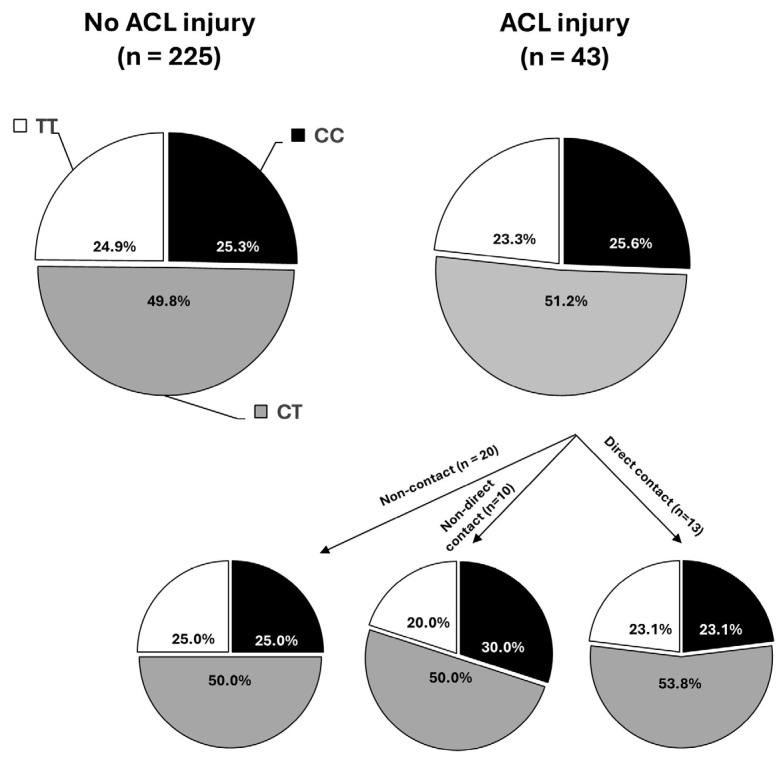
Distribution of the *COL5A1* rs12722 genotype in 268 professional football players competing in *LaLiga* depending on their history of anterior cruciate ligament (ACL) rupture certified by arthroscopy. For those with history of ACL rupture, the distribution of the *COL5A1* rs12722 genotype has been presented depending on the mechanism of injury (non-contact, indirect contact, direct contact).

From the study sample, there were 43 players with history of ACL rupture. Figure 3 depicts the distribution of the *COL5A1* rs12722 genotype in the sample of football players depending on their history of ACL rupture. The distribution of genotypes was similar in players with no history of ACL rupture (CC, 25.3%; CT, 49.8%; and TT, 24.9%) with respect to those with a history ACL rupture certified by arthroscopy (CC, 25.6%; CT, 51.2%; and TT, 23.3%, Figure 3). Overall, the prevalence of players with history of ACL injury was 16.2% for the whole group of CC players (11 out of 68 players), 16.4% for whole group of CT (22 out of 135 players) and 15.2% for the group of TT players (10 out of 66 players; *p* = 0.973). Additionally, the distribution of the *COL5A1* rs12722 genotype was similar in players with a non-contact ACL rupture, with a non-direct contact ACL rupture or with a direct contact ACL injury (*p* = 0.995).

### 3.3. Characteristics of the ACL Ruptures

In the sample of 43 players with history of ACL rupture there were confirmed a total of 49 ACL ruptures, 37 players with one ACL rupture and 6 players two ACL ruptures. There was an association between the *COL5A1* rs12722 genotype and the number of ACL injuries (*p* = 0.020). Of the six players who sustained two ACL ruptures, two were CT, four were TT, and none were CC. This represents an unexpectedly high frequency of TT players experiencing multiple ACL ruptures (*p* < 0.050). From these 6 players, four had their second ACL injury in the contralateral leg with respect to the first ACL injury, while two players had an ACL re-injury in the same leg. Table 3 depicts the characteristics of these 49 ACL ruptures registered depending on the *COL5A1* rs12722 genotype of the player that suffered the injury. The time to return to play was not affected by the *COL5A1* rs12722 genotype (*p* = 0.631) and it was on average 210 ± 45 days. The *COL5A1* rs12722 genotype did not modify the dominance of the leg injured, the moment of the season when the injury occurred, the proportion of match/training ACL injuries, the football-specific action that led to the injury, or the moment of the injury within the match (all *p* > 0.050).

### 3.4. Characteristics of Non-Contact ACL Ruptures

From the total of 49 ACL ruptures reported, 24 were originated with a non-contact mechanism. This corresponded to 16 players with one ACL rupture and 4 players with two ACL ruptures. Table 4 depicts the characteristics of the 24 non-contact ACL ruptures registered depending on the *COL5A1* rs12722 genotype of the player that suffered the injury. The time to return to play was not affected by the *COL5A1* rs12722 genotype (*p* = 0.631) and it average was 211 ± 47 days for the three genotypes. Additionally, the non-contact ACL injuries occurred mostly during the in-season period and during matches without differences between genotypes. However, the *COL5A1* rs12722 genotype affected the dominance of the injured leg (*p* = 0.012), the distribution of injuries while attacking/defending (*p* = 0.012), the type of action that lead to the injury (*p* = 0.047), and the distribution of non-contact ACL injuries depending on the match half (*p* = 0.018) and the minutes played at the moment of the injury (*p* = 0.020). Specifically, all CC players suffered their ACL injury in their dominant leg, and 85.7% of the TT players had the injury in their non-dominant leg (all *p* < 0.050), both representing abnormal frequencies of injury with respect CT players. All CC players had their non-contact ACL ruptures when attacking, while 85.7% of the TT players had their non-contact ACL injury were defending (*p* < 0.050), both representing abnormal frequencies of injury with respect to CT players. Additionally, CC players had a higher-than-expected frequency of injuries when landing and reaching (*p* < 0.050) while TT players had a higher-than-expected frequency of injuries when pressing (*p* < 0.050). Last, CC players had all match injuries in the 2nd half with a higher-than-expected frequency of injuries in the 61-75 min and in the 76-90 min period (all *p* < 0.050). However, TT players had 80% of match injuries in the first half with a higher-than-expected frequency of injuries in the 0-15 min period (*p* < 0.050).

## 4. Discussion

The aim of this study was to examine the association between the *COL5A1* rs12722 polymorphism and the prevalence of ACL rupture in a homogeneous sample of professional male football players. We designed this study to solve inconsistencies of previous studies as the literature on the topic includes predominantly small-scale studies with samples of athletes with ACL ruptures practicing different sports and at different performance levels or active individuals not enrolled in any specific sports practice. In the current study, all participants were professional football players playing in the national football league with the highest indices of international prestige and competitive quality [32]. Additionally, we included the analysis of the situational pattern that led to the injury which is another novelty of this investigation. The present data revealed no significant association of the *COL5A1* rs12722 polymorphism with the prevalence of ACL rupture, as the distribution of the CC/CT/TT genotypes was almost identical in players with and without history of ACL rupture. This outcome generally agrees with several studies [12,16,19] and systematic reviews and meta-analyses [18,33]. However, the first outcome of the present study contrasts with other studies that found an association between *COL5A1* rs12722 polymorphism and the likelihood of suffering an ACL rupture in athletes [17,23,26], and football players [15]. Interestingly, in all the investigations that showed association between the *COL5A1* rs12722 polymorphism and the risk of ACL rupture, it was found that either the TT genotype, or the T allele were negative for the likelihood of suffering this injury, as the presence of players with these genetic variants was overrepresented in the sample of athletes with ACL ruptures. The potential negative phenotypic association of the T allele with ACL injury risk is supported by mechanistic evidence, as the TT genotype is thought to increase the production of type V collagen, which may disrupt fibrillogenesis and compromise the overall collagen structure within the tissue [21]. Additionally, although the current study did not find an increased overall prevalence of ACL rupture in TT players, it is noteworthy that among the 10 TT players who sustained an ACL injury, 4 (40%) experienced a second rupture. In contrast, none of the 11 CC players with an ACL injury suffered a subsequent ACL rupture. Collectively, all this information suggests that the role of the *COL5A1* rs12722 polymorphism on the risk of ACL rupture during sporting activities is uncertain at the moment and more studies with homogeneous samples (i.e., athletes of the same sport) are required to determine the potential negative consequences of the TT genotype, if any.

Furthermore, the results of this study showed that the distribution of the *COL5A1* rs12722 genotypes was similar in players who suffered ACL injuries regardless of the mechanism of occurrence (non-contact, indirect contact, direct contact). A possible explanation for this finding could be to the fact that, in our study, 51% of the ACL injuries (i.e., 25 out of 49) were triggered by contact (direct or indirect) with another player or an object. In the case of contact ACL ruptures, external forces took part in the mechanism of the injury, and it is probable that genetics was less associated with this type of injury. While some previous investigations studied only non-contact ACL injuries [16,25], to the best of our knowledge, this is the first study to investigate the distribution of the *COL5A1* rs12722 polymorphism in relation to different mechanisms of ACL injury in sports. Overall, the findings suggest that the *COL5A1* rs12722 genotype does not have a significant influence on the risk of ACL rupture, regardless of whether the injury occurs through contact or non-contact mechanisms. Additionally, this genotype does not affect the characteristics of ACL ruptures in general, when analysing all mechanisms of injury together (Table 3).

Based on previous reports, non-contact ACL ruptures are considered one of the most common mechanisms of ACL injury in football [30,34]. We performed a sub-analysis of the 24 non-contact ACL ruptures found in this study (Table 4), as these injuries are more likely to be influenced by genetic factors. This is because non-contact ACL injuries are more associated with intrinsic factors of the players as muscle imbalances, excessive joint laxity or reduced range of movement or anatomical predispositions (e.g., narrow intercondylar notch) [35]. In the current investigation, we observed that the characteristics of the non-contact ACL ruptures exhibited a differentiated distribution according to the *COL5A1* rs12772 genotype. Specifically, players with the CC genotype predominantly had non-contact ACL ruptures in their dominant leg during attacking situations, such as landing or catching actions, as well as in the last third of the matches. The high prevalence of non-contact ACL injuries in the last minutes of the match has previously been reported [36] but this is the first study that characterizes this type of injury specifically for players with the *COL5A1* rs12772 CC genotype. This suggests that the accumulation of fatigue throughout the match may increase the risk of non-contact ACL injury. In this regard, it is well known that fatigue can lead to the generation of inadequate commands in the motor cortex [37,38] what could alter lower limb mechanics and negatively impact on the knee joint stability [39]. To this regard, the data of this study suggests that CC players may be more susceptible to the potential effect of fatigue on the risk of non-contact ACL injury. At this stage, this remains a hypothesis that requires further investigation, particularly given the small number of players with non-contact ACL injuries.

In contrast, our results showed that players with the TT genotype predominantly had ACL ruptures in their non-dominant leg during defensive situations, particularly in pressing actions in the early 15 min of the matches. Recently, [30] reported that a significant number of ACL ruptures (22.9%) occurred within the first 15 min of matches, probably due to an inadequate neuromuscular readiness of players. ACL ruptures in the first minutes of the match are likely unrelated to fatigue factors [30] and they may be more associated with excessive joint laxity or ligament weakness [40]. Therefore, this study points towards a tendency of TT players to suffer ACL ruptures while defending and in the first minutes of the match and more associated with neurocognitive errors than with fatigue [34]. Interestingly, players with the CT genotype showed an intermediate profile regarding the characteristics of non-contact ACL ruptures, because the dominant and non-dominant leg were injured with almost equal frequency, and in a mixture of offensive and defensive actions. It is difficult to make comparisons as no previous studies have examined the type of action that originated the non-contact ACL ruptures and their association with the *COL5A1* rs12772 genotype. However, the differential characteristics of the non-contact ACL ruptures depending on the *COL5A1* rs12772 genotype found in this investigation coincide with the progressive TT > CT > CC higher messenger RNA stability associated with the T allele [20] and the progressive TT > CT > CC altered biomechanical properties of ligaments [22]. Collectively, we hypothesize that *COL5A1* rs12722 polymorphism was not associated with the prevalence of ACL rupture in professional football players but influenced several characteristics of the injury that may contribute to ACL injury susceptibility.

This information, although preliminary, may be relevant for sports medicine and strength and conditioning practitioners working in ACL prevention in professional football squads, as the strategies to prevent this type of injury may be different depending on the *COL5A1* rs12722 genotype of the players. Based on the current results, CC players should be physically prepared to improve landing and reaching while in fatigue situations through stability and mobility exercises to cope with the demands of these actions in the last phases of the match. On the other hand, TT players should be prepared to improve their neurocognitive control during defensive actions, particularly through pressing exercises with a special focus on bilateral training to avoid the potential reduction of neuromuscular control of their non-dominant leg. Based on the present results, we can suggest that preventing non-contact ACL ruptures in football players who have a TT genotype could be more complex than for players with CC and CT genotypes, whose injuries may be simpler to prevent due to the potential key role of fatigue as a contributing factor. In fact, previous studies have observed how the workload control, in football players to avoid fatigue [41,42,43], as well as neuromuscular training decreases the risk of non-contact injuries [44]. Finally, suggestions for preventing ACL injury based on the *COL5A1* rs12722 genotype in football players remain subject to validation in future research, as the observed association with non-contact ACL ruptures—those potentially influenced by genetic factors—does not imply causality.

Several potential limitations should be acknowledged in the present investigation. Firstly, the study population consisted of a specific sample of 268 male professional football players, of whom only 43 had sustained an ACL injury. Among these, just 20 players had non-contact ACL tears, the ones more potentially associated with genetics. This relatively low ratio is expected given the overall low prevalence of ACL injuries in football. Although we successfully recruited players from half of the teams competing in *LaLiga*, the sample size of players with ACL injuries, particularly those with non-contact mechanisms, may still be considered limited. Additionally, the present findings may therefore not be readily extended to e.g., younger players, female football players nor to the general population as a whole. Secondly, it is key to note that our analyses only considered ACL ruptures while other injuries such as meniscus tears or other knee ligament sprains during the same action were not included. Third, the results of this investigations are specific for a single SNP however, there are other SNPs in the *COL5A1* genotype such as the rs13946 that could have also a role in the risk of ACL rupture. In fact, some studies have found that the haplotype TT as consequence of the combination of rs12722 and rs13946 genetic variants is the combination that ultimately increases the risk of injury while possessing only the T allele or the TT genotype in only one of these variants is not enough to increase the potential risk of ACL injury [25]. Fourth, while this study focused on the association between the *COL5A1* rs12722 polymorphism and ACL injury prevalence in football players, it is important to acknowledge that gene expression and ligament structure are not solely determined by players’ genetic sequence. Epigenetic mechanisms, such as DNA methylation, histone modification, and non-coding RNA regulation may also play a key role in modulating *COL5A1* expression and collagen synthesis. These mechanisms can be influenced by environmental and lifestyle factors, including training load, nutrition, sleep, psychological stress, and injury history. Therefore, interindividual differences in ACL injury susceptibility may not be fully captured by the analysis of DNA alone. Future studies should aim to integrate genetic and epigenetic data, potentially using multi-omics approaches, to better understand the complex biological pathways underlying ligament injuries in professional football players. Finally, the players examined are part of 10 different teams, each of which has been exposed to different workloads and specific training programs, and they may have different ACL tear prevention programs. Additionally, the accumulated training and match time over the season is an exclusive decision of the coach staff of each team. So, this study did not completely fulfil the criteria of exchangeability required for strengthening outcomes of observational research in genetics [45].

## 5. Conclusions

**Figure 4:**
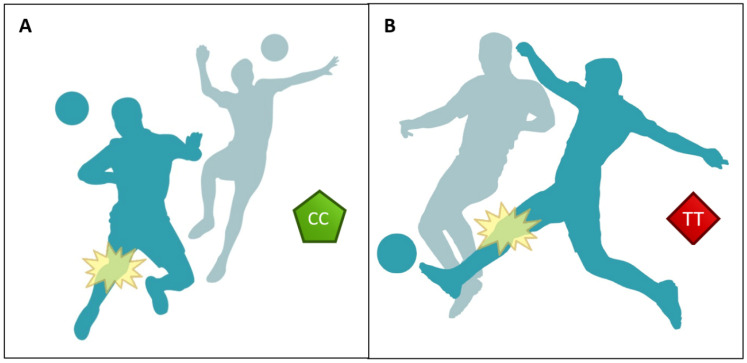
Representative image of the effect on the *COL5A1* rs12722 polymorphism on the conditions under which ACL injuries occur in professional football players. Players with the CC genotype predominantly sustained non-contact ACL injuries during attacking situations and in the latter stages of a match (**A**). Players with the TT genotype were more likely to experience non-contact ACL injuries while pressing and in the early minutes of a match (**B**).

In summary, the *COL5A1* rs12722 polymorphism was not associated with the prevalence of ACL rupture in a sample of 268 professional football players competing in *LaLiga*. Overall, the prevalence of players with history of ACL injury, with respect to the total number of players within each genotype, was 16.2% for CC, 16.4% for CT and 15.2% for TT players. This indicates that, in this sample of professional football players, none of the variants in the *COL5A1* rs12722 polymorphism were considered protective/negative for the likelihood of suffering this type of ligament injury during football exposure. Additionally, the *COL5A1* rs12722 polymorphism showed no association with the characteristics of ACL ruptures, likely because 51% of ACL ruptures reported were associated with contact mechanisms (either indirect or direct) which are not potentially associated with genetics. However, the analysis of the sub-group of non-contact ACL ruptures (the ones potentially affected by genetics) demonstrated that the characteristics of the ACL tears were associated with the *COL5A1* rs12722 genotype. *COL5A1* CC players predominantly had non-contact ACL injuries in their dominant leg, while landing or reaching in attacking situations, and during the last phases of the match. On the contrary, *COL5A1* TT players had predominantly non-contact ACL injuries in their non-dominant leg, while pressing an opponent, and during the initial minutes of the match (Figure 4). These findings reinforce that ACL ruptures are multifactorial injuries, with numerous intrinsic and extrinsic contributors. While the *COL5A1* rs12722 polymorphism may play a role in shaping specific injury characteristics, its overall contribution appears limited. Importantly, many of the risk factors associated with ACL injury, such as neuromuscular control, fatigue, and movement patterns are modifiable through targeted and individualized training programs.

## Figures and Tables

**Table 1:** Terms and categories used to describe the mechanism and situational patterns of anterior cruciate ligament (ACL) ruptures occurred in professional male football players.

Variable	Categories
Injury mechanism	Non-contact; indirect contact; direct contact
Injured limb	Dominant; non-dominant
First vs. second injury	First injury; second injury in the same leg; second injury in the contralateral leg
Season phase	Preseason; In-season
Type of exposure	Match; training
Context	Attacking; defending
Action	Tackling/being tackled; landing; cutting; kicking; pressing; reaching; other
Match injury time	1st half; 2nd half
Minutes played	00-15; 16-30; 31-45; 46-60; 61-75; 76-90

**Table 2:** Sociodemographic variables in 268 professional football players competing in *LaLiga* depending on their *COL5A1* rs12722 genotype.

Variable (Units)	CC	CT	TT	*p* Value
**Sociodemographic variables**				
Number [frequency (%)]	68 (25.4)	134 (50.0)	66 (24.6)	-
Age (years)	26.8 ± 4.8	26.8 ± 4.6	25.9 ± 4.6	0.362
Height (cm)	183 ± 6	181 ± 6 *	181 ± 6	0.027
Body mass (kg)	77.8 ± 6.6	75.7 ± 6.4	76.7 ± 6.9	0.133
Body mass index (kg/m^2^)	22.9 ± 3.1	22.8 ± 3.2	23.4 ± 1.5	0.405
**Ethnicity**				
Caucasian [frequency (%)]	57 ↓ (23.1)	128 (51.8)	62 (25.1)	0.037
Afro-American [frequency (%)]	10 ↑ (50.0)	6 (30.0)	4 (20.0)	
Asian [frequency (%)]	1 (100.0)	0 (0.0)	0 (0.0)	
**Field position**				
Goalkeeper	9 (13.2)	16 (11.9)	3 (4.5)	0.107
Center defender	11 (16.2)	16 (11.9)	17 (25.8)	
Full back	13 (19.1)	24 (17.9)	8 (12.1)	
Central midfielder	18 (26.5)	40 (29.9)	18 (27.3)	
Forward	11 (16.2)	25 (18.7)	7 (10.6)	
Winger	6 (8.8)	13 (9.7)	13 (19.7)	

* Different from CC players at *p* < 0.050 for continuous variables analysed with a one-way ANOVA (Bonferroni correction). (↓↑) The value was different from the frequency expected for categorical variables analysed with Chi-square tests (standardized residuals) at *p* < 0.050.

**Table 3:** Characteristics of 49 anterior cruciate ligament (ACL) ruptures registered in 43 professional football players competing in *LaLiga* depending on their *COL5A1* rs12722 genotype.

Variable	CC	CT	TT	*p* Value
Return to play (days)	210 ± 66	211 ± 49	202 ± 33	0.631
Dominant leg	81.8%	58.3%	35.7%	0.068
Non-dominant leg	18.2%	41.7%	64.3%	
Pre-season	9.1%	12.5%	7.1%	0.862
In-season	90.9%	87.5%	92.9%	
Match	90.9%	100.0%	78.6%	0.066
Training	9.1%	0.0%	21.4%	
Attacking	63.6%	41.7%	42.9%	0.451
Defending	36.4%	58.3%	57.1%	
Tackling/tackled	9.1%	25.0%	35.7%	0.055
Landing	27.3%	4.2%	0.0%	
Cutting	0.0%	25.0%	14.3%	
Kicking	27.3%	8.3%	7.1%	
Pressing	9.1%	20.8%	42.9%	
Reaching	18.1%	8.3%	0.0%	
Other	9.1%	8.3%	0.0%	
1st half	50.0%	41.7%	36.4%	0.816
2nd half	50.0%	58.3%	63.6%	
0–15	0.0%	12.5%	18.2%	0.626
16–30	30.0%	16.7%	0.0%	
31–45	20.0%	12.5%	18.2%	
46–60	10.0%	37.4%	36.3%	
61–75	10.0%	4.2%	9.1%	
76–90	30.0%	16.7%	18.2%	

Note: The distribution of injuries in 1st vs. 2nd part or in the different time intervals of the match was only calculated. There were 49 ACL injuries among 43 football players, as 6 players sustained two ruptures during the study period.

**Table 4:** Characteristics of 24 anterior cruciate ligament (ACL) ruptures with a non-contact mechanism registered in 20 professional football players competing in *LaLiga* depending on their *COL5A1* rs12722 genotype.

Variable	CC	CT	TT	*p* Value
Return to play (days)	204 ± 20	218 ± 61	203 ± 33	0.758
Dominant leg	100.0% ↑	41.7%	14.3% ↓	0.012
Non-dominant leg	0.0% ↓	58.3%	85.7% ↑	
Pre-season	0.0%	8.3%	14.3%	0.677
In-season	100.0%	91.7%	85.7%	
Match	80.0%	100.0%	71.4%	0.163
Training	20.0%	0.0%	28.6%	
Attacking	100.0% ↑	41.7%	14.3% ↓	0.012
Defending	0.0% ↓	58.3%	85.7% ↑	
Tackling/tackled	0.0%	0.0%	0.0%	0.047
Landing	40.0% ↑	8.3%	0.0% ↓	
Cutting	0.0%	41.8%	28.6%	
Kicking	20.0%	8.3%	0.0%	
Pressing	0.0%	33.3%	71.4% ↑	
Reaching	40.0% ↑	8.3%	0.0%	
Other	0.0%	0.0%	0.0%	
1st part	0.0% ↓	75.0%	80.0%	0.018
2nd part	100.0% ↑	25.0%	20.0%	
0–15	0.0%	16.7%	60.0% ↑	0.020
16–30	0.0%	33.3%	0.0%	
31–45	0.0%	25.0%	20.0%	
46–60	0.0%	16.7%	20.0%	
61–75	25.0% ↑	0.0%	0.0%	
76–90	75.0% ↑	8.3%	0.0%	

Note: The distribution of injuries in 1st vs. 2nd part or in the different time intervals of the match was only calculated for the ACL ruptures that occurred during a competitive match (n = 21). (↓↑) The value was different from the frequency expected for categorical variables analysed with Chi-square tests (standardized residuals) at *p* < 0.050. There were 24 ACL injuries among 20 football players, as 4 players sustained two ruptures during the study period. Each ACL injury was analyzed independently for these players.

## Data Availability

The data presented in this study are available on request from the corresponding authors, due to restrictions intended to protect the identity of the participating players.

## Abbreviations

The following abbreviations are used in this manuscript:

ACL	Anterior cruciate ligament
COL5A1	Gene codifying the α1 chain of type V collagen
DNA	Deoxyribonucleic Acid
FIFA	Fédération Internationale de Football Association
HWE	Hardy-Weinberg Equilibrium
MRI	Magnetic Resonance Imaging
PCR	Polymerase Chain Reaction
UEFA	Union of European Football Associations
